# Analysis of furans and pyridines from new generation heated tobacco product in Japan

**DOI:** 10.1186/s12199-021-01008-1

**Published:** 2021-09-13

**Authors:** Kanae Bekki, Shigehisa Uchiyama, Yohei Inaba, Akira Ushiyama

**Affiliations:** Department of Environmental Health, National Institute of Public Health, 2-3-6 Minami, Wako, Saitama, 351-0197 Japan

**Keywords:** Heated tobacco product, Pyridines, Furans, Sorbent cartridge

## Abstract

**Background:**

In recent years, heated tobacco products (HTPs), which are widely used in Japan, have been sold by various brands using additives such as flavors. It has been reported that the components of mainstream smoke are different from those of conventional cigarettes. In this study, we established an analytical method for furans and pyridines in the mainstream smoke, which are characteristic of HTPs and particularly harmful among the generated components, and investigated the amount of component to which the smokers are exposed.

**Methods:**

We established a simple analytical method for simultaneous analysis of gaseous and particulate compounds in the mainstream smoke of HTPs (IQOS, glo, ploom S) in Japan by combining a sorbent cartridge and glass fiber filter (Cambridge filter pad (CFP)). Both the sorbent cartridge and CFP were extracted using 2-propanol and analyzed via GC-MS/MS to determine the concentration of furans and pyridines generated from each HTP.

**Results:**

The results showed that the levels of target furans such as furfural, 2-furanmethanol, 2(5H)-furanone, and 5-methylfurfural tended to be higher in the mainstream smoke of glo than in standard cigarettes (3R4F). Pyridine, which is generated at a high level in 3R4F as a combustion component, and 4-ethenylpyridine (EP), which is a known marker of environmental tobacco smoke, were detected. Among these components, 2-furanmethanol and pyridine are classified as Group 2B (possibly carcinogenic to humans) by the International Agency for Research on Cancer (IARC). Therefore, it is possible that they will contribute to the health effects caused by use of HTPs.

**Conclusions:**

Using the new collection and analytical method for furans and pyridines in the mainstream smoke of HTPs, the level of each compound to which smokers are exposed could be clarified. By comprehensively combining information on the amount of ingredients and toxicity, it will be possible to perform a more detailed calculation of the health risks of using HTPs. In addition, the components detected in this study may be the causative substances of indoor pollution through exhaled smoke and sidestream smoke; therefore, environmental research on the chemicals generated from HTPs would be warranted in future studies.

**Supplementary Information:**

The online version contains supplementary material available at 10.1186/s12199-021-01008-1.

## Background

In recent years, much interest has been focused on new types of cigarettes, such as heated tobacco products (HTPs), which are widely used in Japan. The main feature of this new type of cigarette is the reduced content of harmful compounds compared to that in other conventional cigarettes. However, as these products are available on the market, there are few epidemiological data on carcinogenicity, genotoxicity, and the like, and many unknown problems remain regarding toxicity and safety. In fact, carcinogenic compounds such as tobacco-specific nitrosamines (TSNAs) [1] and carbonyl compounds [2], which are regulated by WHO, have been detected in the mainstream smoke of HTPs so far. The concentration of these compounds was significantly reduced compared to that in cigarettes. Although the concentration of these hazardous compounds is very low, smokers are at risk of direct exposure to these substances and face health risks. Furthermore, among the components detected in the mainstream smoke of HTPs, those with a concentration similar or higher than that in cigarettes have also been detected [3].

Among these detected components, additives such as flavors and compounds generated due to heating of the additives tend to be detected at relatively high concentrations compared to other combustion materials. In general, most of the additives used as flavors in tobacco products are safe as food additives, but the American Food Fragrance Manufacturers Association (FEMA) has pointed out that some of these food additives are of concern for safety (1037 types) [4]. In addition, among the detected components, furans including 2(5H)-furanone and 2-furanmethanol, which are also detected in foods after thermal decomposition of sugars, and pyridines (e.g., 4-ethenylpyridine (4-EP), which is produced following thermal decomposition of nicotine), were also detected in the smoke of HTPs [3]. Among these components, 2-furanmethanol and pyridine are classified as Group 2B (possibly carcinogenic to humans) by the International Agency for Research on Cancer (IARC). In addition, 2(5H)-furanone has also been reported to have toxic effects such as cytotoxicity due to DNA fragmentation in in vitro studies [5], and benzyl alcohol, linalool, menthol, and eugenol have also been used for the purpose of flavor [3]. It has also been suggested that these components are related to respiratory diseases, and their toxicity mechanism has been reported to involve an agonist compound of TRP channel (TRPA1), which is an inducer of cytotoxicity [6]. Therefore, it is possible that various additives contribute to health risks. However, data on these compounds are very limited, and there are few data on domestic HTPs [3]. In this study, we established a simple analytical method for simultaneous analysis of gaseous and particulate compounds by combining a sorbent cartridge and CFP; the method is an individual method for the collection of furans and pyridines from the mainstream smoke of HTPs. Furthermore, we tried to determine the amount of components generated from each HTP and the levels of the components to which smokers are exposed to obtain basic data for investigating their effects on human health.

## Methods

### Apparatus and reagents

Gas chromatography (GC) coupled with a triple quadrupole mass spectrometer (GCMS-TQ8040, SHIMADZU) and HP-5MS (30 m × 0.25 mm i.d., 0.25 μm) (Agilent Technologies, CA, USA) were used to quantify furans, pyridines, and other additives in the mainstream smoke in this study. GC coupled with a thermal conductivity detector (TCD) and a packed column (Porapack Q2 m×3 m i.d., 80–100 mesh deactivated stainless, GL Sciences, Tokyo, Japan) was used to quantify water in the mainstream smoke in this study. Nicotine 97%, n-hexane > 96%, 2-propanol > 99.7%, methanol (high-performance liquid chromatography [HPLC] grade) > 99.9%, acetic acid > 99.7%, and 2,6-dimethylpyridine were purchased from FUJIFILM Wako Chemicals, Ltd. (Osaka, Japan). 2,5-Dimethylpyrazine, 2(5H)-furanone, 5-methylfurfural, nicotine, acetonitrile, and ammonium acetate (≥ 99.99%) were purchased from Sigma-Aldrich Inc. (St. Louis, MO, USA). Pyridine, furfural, 2-furanmethanol, 2-ethenylpyridine (2-EP), 3-ethenylpyridine (3-EP), 4-ethenylpyridine (4-EP), 3-ethyl pyridine, 2,3,5-trimethylpyrazine, benzyl alcohol, linalool, menthol, 5-hydroxy-2-methylpyridine, isoquinoline, eugenol, and 4-ethyl guaiacol were purchased from Tokyo Kasei Co., Ltd., (Tokyo, Japan). The water used for sample preparation and analysis was deionized, and it was further purified using a Milli-Q water system (Millipore Co., Bedford, MA, USA).

In this study, we used conventional combustion cigarettes (3R4F) from the University of Kentucky (Lexington, KY, USA), IQOS (regular and menthol) from Philip Morris International Inc. (NY, USA), glo (berry boost and dark fresh) from British American Tobacco (NC, USA), and ploom S from Japan Tobacco Inc. (Tokyo, Japan). According to the International Organization for Standardization (ISO) 3402, these cigarettes were used for measurement after being placed at 22°C and 60% humidity for 2 days [7].

### Method of sampling main stream smoke of HTPs

For sampling of the gas phase and particle phase of mainstream smoke of HTPs, a sorbent cartridge and CFP were used. To prepare the sorbent cartridge, 150 mg of Tenax GR (GL Sciences, Tokyo, Japan) was packed in a Rezorian tube (1 mL), and the cartridge was washed with 10 mL of 2-propanol and dried with nitrogen gas at a flow rate of 1.0 L/min for 5 min. Mainstream smoke of conventional combustion cigarette was collected with this sorbent cartridge connected between tobacco product and CFP according to the intense regime described in the standard operating procedure (SOP) 01 [8] and Health Canada, official method T-115 [9]. Briefly, mainstream smoke was collected under the following conditions: 55 mL puff volume, 2 s puff duration, 30 s puff interval, and 100% blocking of the filter ventilation holes with Mylar adhesive tape. The puff number of 3R4F as a standard conventional combustion cigarette was nine times. In case of HTPs, we produced a puffing profile for the collection of mainstream smoke of HTPs from battery time conditions of HTPs as described below. The maximum number of smoking absorption was twelve times for one dedicated stick from the calculation of smoking interval based on the HCI method (30 s) and battery time of IQOS (6 min). When the number of puffs was calculated for glo and ploom S by the same method as IQOS, the number of puffs for IQOS was the largest. Therefore, in order to compare the concentrations of three types of HTPs in the mainstream smoke, the total number of puffs was set as twelve puffs. So far, no official law has been established regarding the smoking interval and amount of smoke absorbed for HTPs. Actually, it is possible that research on smoking behavior of smokers who use HTPs has not been sufficiently conducted. Therefore, it is necessary to consider it as a future issue. Furthermore, for volatile compounds, many of them pass through the CFP at the time of collection and are collected in the sorbent cartridge, but those collected in the CFP may be slightly volatile during the collection of mainstream smoke of HTPs. Therefore, we need to do quickly pretreatment after the sample collection.

### Analytical method

Both the sorbent cartridge and CFP were extracted using 5 mL and 10 mL of 2-propanol, respectively, for 30 min using a rotary shaker at 120 cycles per min. After extraction, isoquinoline was added as an internal standard and analyzed via GC-MS/MS. The GC oven temperature program was 50 °C (held for 2 min) and increased to 200 °C at 6 °C/min and 310 °C (held for 3 min) at 20 °C/min. Splitless injections were carried out at an injector temperature of 280 °C and splitless time of 1 min. Helium (99.99995%) was used as the carrier gas at a flow rate of 1.3 mL/min (pressure 75 kPa). Mass spectrometric ionization was performed in the electron impact (EI) mode at a voltage of 70 eV. The transfer line and ion source temperatures were set to 280 °C and 230 °C, respectively. To identify suitable precursor ions, full scan monitoring was performed for all compounds at the first MS with a mass range of *m*/*z* 50–500. Argon (99.999%) was used as the collision-induced dissociation (CID) gas. To determine suitable product ions, full product ion spectra of selected precursor ions were obtained, and the optimum collision energies were determined for each identified *m*/*z* transition. The most abundant and apparent ions were chosen as the targets for quantification. The peaks of each compound that showed a signal-to-noise ratio (S/N) of 3 in the sample were identified by comparing the retention times (within 0.1 min) and the ratios (within 20%) of the two selected reaction monitoring transitions in the sample with those of the standards.

For the analysis of tar, we referred to the previous report by Bekki et al. [1]. The amount of tar exhausted in the mainstream smoke was calculated by subtracting the amount of nicotine and water from the total particulate matter (TPM) collected by CFP.

## Results and discussion

### Sample collection and pretreatment

**Fig. 1:**
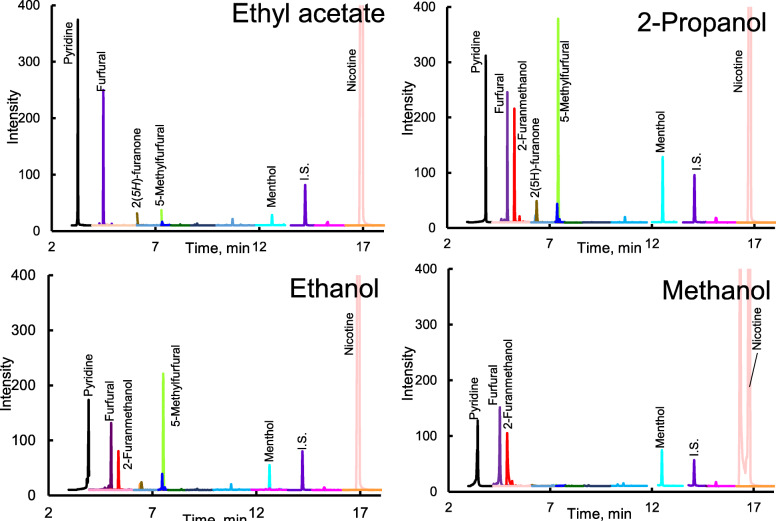
MRM chromatograms of mainstream smoke of IQOS (regular) extracted with polar and non-polar solvent

To examine the extraction conditions of the components collected in the sampler, the extraction efficiency was investigated using organic solvents with different polarities as follows (2-propanol < ethyl acetate < ethanol < methanol). Because the physical properties regarding the polarity of each component are different, the extraction efficiency may differ depending on the type of solvent. Figure 1 shows the chromatograms obtained for the four different types of solvents. The detection sensitivity was slightly different for each component and solvent, and most of the components were detected more sensitively by 2-propanol which has properties as a polar solvent because it has hydrogen bonding properties due to hydroxy groups, while it also has properties as a non-polar solvent because it also has an isopropyl group which is a hydrophobic group. Therefore, 2-propanol is considered to have comprehensive solubility even for components having different polarities, and we decided to use 2-propanol as the extraction solvent.

To determine the recovery rate of the compounds collected in the Tenax GR cartridge and CFP, 5 μg of 19 targeting compounds was added to this cartridge and dried with nitrogen gas for 5 min at a flow rate of 1 L/min. After adding 5 mL of 2-propanol, extraction was performed using a rotary shaker at 120 cycles per min for 30 min. The eluate was analyzed using GC-MS/MS. The recovery rate of 2-propanol was in the range of 91–102% for the Tenax GR cartridge.

### Calibration curve, LOD, and LOQ

To obtain the quality control protocol for the analysis of furans, pyridines, and other additives, calibration curves were generated from the ratios of the areas of the peaks of six known concentrations of standard and quinoline as an internal standard. The detection limit (LOD) and quantification limit (LOQ) for the measurement of furans and pyridines were defined as three and ten times the standard deviation of the lowest standard, respectively. Each calibration curve showed good linearity (*r*^*2*^ > 0.9772) in the range of 0.005–0.2 μg/mL for 2,6-dimethylpyridine and 2(5H)-furanone; 0.01–0.5 ng/mL for 5-methylfurfural, pyridine, 2,5-dimethylpyrazine, and 2-EP; 0.002–0.5 ng/mL for 3-ethylpyridine; 0.01–1.0 μg/mL for methanol and 4-ethyl guaiacol; 0.02–1.0 μg/mL for furfural, 3-EP, 4-EP, and 2,3,5-trimethylpyrazine; 0.5–50 μg/mL for 2-furanmethanol; and 0.05–1.0 μg/mL for nicotine, benzyl alcohol, and eugenol (Table S1).

### Concentrations of furans and pyridines in the mainstream smoke of HTPs

**Fig. 2:**
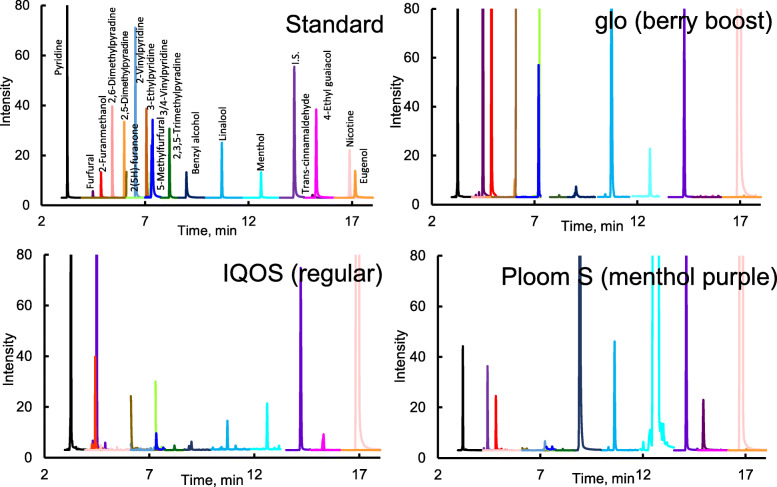
MRM chromatograms of standard and mainstream smoke of HTPs (IQOS, glo, ploom S). The concentration of the standard mix was 0.5 μg/mL

Figure 2 shows an MRM chromatogram obtained from the analysis of furans, pyridines, and other target compounds from the mainstream smoke extract of IQOS (regular). At this time, 12 of the 19 target compounds were detected. These components in the mainstream smoke of HTPs mainly include components produced by heating and migrated from tobacco leaves. In addition, the concentration shown in Table 1 is the sum of the components detected using the sorbent cartridge and CFP.

**Table 1:** Concentrations of chemical compounds detected in the mainstream smoke of HTPs using Tenax GR cartridge and CFP (μg/stick) (*n* = 3). n.d. means not determined

Compound (μg/stick)	IQOS		glo		ploom S		3R4F
	Regular	Menthol	Berry boost	Dark fresh	Regular taste	Menthol purple	
***Furans***							
Furfural	98 ± 16	1.7 ± 0.17	170 ± 8.4	180 ± 16	11 ± 0.49	2.0 ± 0.16	86 ± 22
2-Furanmethanol	15 ± 5.1	0.11 ± 0.010	34 ± 4.5	46 ± 5.6	2.4 ± 0.18	2.1 ± 0.11	4.2 ± 0.74
2(5*H*)-Furanone	3.0 ± 0.98	n.d.	12 ± 1.8	14 ± 1.1	1.4 ± 0.10	n.d.	6.6 ± 0.91
5-Methylfurfural	33 ± 6.8	0.35 ± 0.030	55 ± 4.3	70 ± 8.2	2.7 ± 0.17	1.0 ± 0.07	12 ± 2.3
***Pyridines***							
Pyridine	5.2 ± 0.84	6.1 ± 0.85	2.7 ± 0.33	3.1 ± 0.20	0.75 ± 0.020	0.41 ± 0.02	33 ± 4.1
2,6-Dimethylpyridine	n.d.	n.d.	n.d.	n.d.	n.d.	n.d.	1.5 ± 0.28
2,5-Dimethylpyrazine	0.78 ± 0.013	0.010 ± 0.0	0.42 ± 0.030	0.53 ± 0.0	0.73 ± 0.020	0.22± 0.03	4.0 ± 0.62
2-Ethenylpyridine	n.d.	n.d.	n.d.	n.d.	n.d.	n.d.	0.59 ± 0.10
3-Ethylpyridine	n.d.	n.d.	n.d.	n.d.	n.d.	n.d.	1.4 ± 0.93
4-Ethenylpyridine	1.5 ± 0.30	0.67 ± 0.14	2.5 ± 0.26	0.94 ± 0.030	0.65 ± 0.010	1.1 ± 1.0	6.3 ± 1.4
3-Ethenylpyridine	0.50 ± 0.13	n.d.	0.15 ± 0.010	0.55 ± 0.080	0.31 ± 0.020	0.19 ± 0.060	3.7 ± 0.36
2,3,5-Trimethylpyrazine	n.d.	n.d.	n.d.	n.d.	n.d.	n.d.	n.d.
***Additives***							
Benzyl alcohol	0.30 ± 0.080	0.080 ± 0.010	0.62 ± 0.12	1.0 ± 0.050	0.22 ± 0.020	n.d.	4.2 ± 0.31
Linalool	0.52 ± 0.36	0.020 ± 0.0	13 ± 1.4	0.05 ± 0.010	0.020 ± 0.0	3.4 ± 0.18	0.12 ± 0.11
Menthol	2.5 ± 0.22	1100 ± 64	1700 ± 200	1500 ± 41	0.93 ± 0.18	720 ± 12	4.5 ± 3.9
4-Ethyl guaiacol	n.d.	n.d.	n.d.	n.d.	n.d.	n.d.	0.79 ± 0.090
Eugenol	n.d.	n.d.	n.d.	n.d.	n.d.	0.10 ± 0.01	n.d.
***Others***							
Nicotine (mg/stick)	1.2 ± 0.059	1.3 ± 0.082	1.0 ± 0.0037	1.2 ± 0.022	0.7 ± 0.0	0.6 ± 0.0	1.8 ± 0.0
Water (mg/stick)	27 ± 7.7	26 ± 4.4	15 ± 1.0	17 ± 1.7	16 ± 0.3	18 ± 0.6	10 ± 2.3
Tar (mg/stick)	13 ± 3.0	10 ± 5.3	22 ± 2.4	18 ± 2.5	13 ± 8.5	12 ± 9.8	27 ± 1.8
Total (mg/stick)	42 ± 1.3	38 ± 0.42	38 ± 2.3	37 ± 2.8	30 ± 3.3	31 ± 3.3	49 ± 3.7

Among the target components detected from IQOS, furans were detected at relatively high concentrations in the following order: furfural > 5-methylfurfural > 2-furanmethanol > 2(5H)-furanone, and pyridine was also detected at high concentration. And, almost of these components were detected in the gas phase collected by sorbent cartridge (Tables S3, S4, S5 and S6). These furans are often derived from flavors used for HTPs; however, conversely, they are also known as a product of heating reaction of sugars and amino acids contained in tobacco leaves, which is known as a Maillard reaction [10–12]. Furthermore, EP has generally been used as a gaseous marker of environmental tobacco smoke (ETS) derived from nicotine. Therefore, it was considered that the EP detected here could also be used as a marker component of the second- and third-hand smoke of HTPs [13, 14]. In fact, some volatile compounds in the mainstream smoke of cigarettes and electronic cigarettes are emitted from the exhaled breath of smokers [15, 16]. A few studies have also reported the sidestream emission of some volatile compounds, including EP [17]. Therefore, it is possible that EP will become an evaluation index for the effects of exhaled smoke and second- and third-hand smoke of HTPs in an indoor environment. However, EP cannot be used as an environmental marker only for HTPs because it is also affected by cigarettes.

In addition, the mainstream smoke of three types of HTPs (IQOS, glo, and ploom S) that are in high demand in Japan was compared in this study. As shown in Table 1, similar to IQOS, relatively large amount of furans was detected, especially in glo and ploom S. The reason for these results is that furans are widely used as fragrances (furfural, 5-methylfurfural, 2-furanmethanol) in HTPs. Therefore, it was presumed that relatively high concentrations would be detected in glo, which has many brands that use various flavors. In contrast, pyridine and nicotine were detected at relatively higher concentrations in IQOS than in glo and ploom S, although these concentrations were significantly lower than the cigarette (3R4F) burned at around 800 °C. The reason for these results was speculated to be the difference in the heating temperature of each product: IQOS (350 °C) > glo (240 °C) > ploom S (200 °C). Furthermore, the principle of each HTPs is very different. Actually, IQOS use a stick containing tobacco leaves which is inserted into a holder and heated from the inside with a heating blade. On the other hand, glo and ploom S use a stick containing tobacco leaves inserted into a holder and heated from the surroundings. These characteristics also may affect the difference of concentrations of each compound detected in the mainstream smoke of HTPs.

### Amount of target compounds detected in the mainstream smoke of HTPs with the puff numbers

**Fig. 3:**
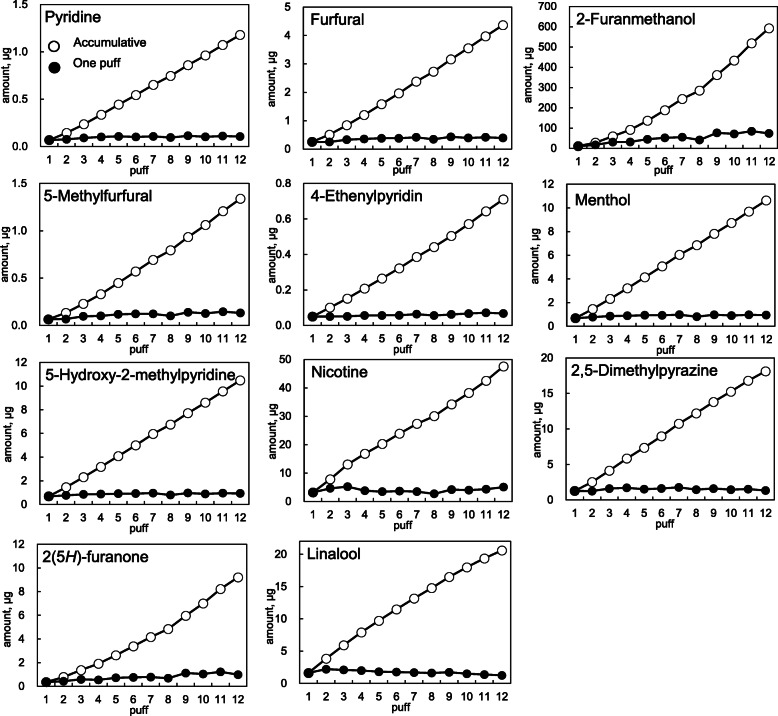
Changes in the amount of target compounds detected in the mainstream smoke of IQOS (regular)

Figure 3 shows the concentration of furans and pyridines in mainstream smoke generated from IQOS (regular) using a sorbent cartridge for each puff. In this figure, the amount of component collected from each puff is indicated with a white marker, and the value accumulated up to 12 puffs is indicated with a black marker. From these results, it was confirmed that most of the compounds increased depending on the number of puffs. However, 2-furanmethanol was below the detection limit up to the second puff, and it seemed that it would take time from heating to generation. In addition, in this study, three heat sticks were used for the collection and quantification of compounds emitted from the mainstream smoke of HTPs in one sorbent cartridge. However, the results of this experiment showed that it was possible to quantify the components in mainstream smoke by the number of puffs or by one heat stick. In fact, the official law regarding the smoking method of HTPs has not been established because the smoking behavior of smokers who use HTPs has not been clarified. Therefore, it is also possible to collect and quantify the mainstream smoke suitable for the smoking behavior of HTPs.

## Conclusions

By using the sorbent cartridge and CFP for the collection and analysis of furans and pyridines in the mainstream smoke of HTPs, the level of each compound to which smokers are exposed could be determined. By comprehensively combining information on the amount of ingredients and toxicity, it will be possible to perform a more detailed calculation of health risks compared to that performed before. Furthermore, in recent years, the effects of indoor pollution and second-hand smoke caused by HTPs have been reported. Therefore, the compounds detected at relatively high concentrations of HTPs in this study may be used as indicators of second- and third-hand smoke in the future studies.

## Supplementary Information

**Additional file 1: Supplementary data.** Supplementary data associated with this article can be found in the table S1, S2. **Table S1.** Calibration curves, detection limits, and quantification limits for the chemical compounds. **Table S2.** MS/MS parameters for the target compounds. **Table S3.** Concentrations of chemical compounds detected in the mainstream smoke of IQOS using Tenax GR cartridge and CFP (μg/stick) (*n=*3). n.d. means not determined. **Table S4.** Concentrations of chemical compounds detected in the mainstream smoke of glo using Tenax GR cartridge and CFP (μg/stick) (*n=*3). n.d. means not determined. **Table S5.** Concentrations of chemical compounds detected in the mainstream smoke of ploom S using Tenax GR cartridge and CFP (μg/stick) (*n=*3). n.d. means not determined.

## Data Availability

Please contact the author for data requests.

## Abbreviations

CFP: Cambridge filter pad
CID gas: Collision-induced dissociation gas
EI: Electron impact
3-EP: 3-Ethenylpyridine
4-EP: 4-Ethenylpyridine
FEMA: American Food Fragrance Manufacturers Association
GC-MS/MS: Gas chromatography coupled with triple quadrupole mass spectrometer
GC-TCD: Gas chromatography coupled with a thermal conductivity detector
HTPs: Heated tobacco products
HPLC: High-performance liquid chromatography
IARC: International Agency for Research on Cancer
ISO: International Organization for Standardization
LOD: Detection limit
LOQ: Quantification limit
SOP: Standard operating procedure
TSNAs: Tobacco-specific nitrosamines
