# Supplementary material for: Analysis of furans and pyridines from new generation heated tobacco product in Japan
Source: Environ Health Prev Med. 2021 Sep 13;26:89. doi: 10.1186/s12199-021-01008-1 (PMC8438973; doi:10.1186/s12199-021-01008-1)
Supplement: Supplementary file 1 — Additional file 1: Supplementary data. Supplementary data associated with this article can be found in the table S1, S2. Table S1. Calibration curves, detection limits, and quantification limits for the chemical compounds. Table S2. MS/MS parameters for the target compounds. Table S3. Concentrations of chemical compounds detected in the mainstream smoke of IQOS using Tenax GR cartridge and CFP (μg/stick) (n=3). n.d. means not determined. Table S4. Concentrations of chemical compounds detected in the mainstream smoke of glo using Tenax GR cartridge and CFP (μg/stick) (n=3). n.d. means not determined. Table S5. Concentrations of chemical compounds detected in the mainstream smoke of ploom S using Tenax GR cartridge and CFP (μg/stick) (n=3). n.d. means not determined. [file 12199_2021_1008_MOESM1_ESM.docx]

Supplementary material

for

**Analysis of furans and pyridines from new generation**

**heated tobacco product in Japan**

Kanae Bekki*, Shigehisa Uchiyama, Yohei Inaba, Akira Ushiyama

Department of Environmental Health, National Institute of Public Health, 2-3-6 Minami, Wako, Saitama 351-0197, Japan

*Corresponding author: Kanae Bekki, E-mail: bekki.k.aa@niph.go.jp, Tel.: +81-48-458-6258; Fax: +81-48-458-6270.

Table S1

| Compound | Range (µg/ml) | Regression | Coefficient of determination (*r^2^*) | LOD (ng/ml) | LOQ (ng/ml) |
| --- | --- | --- | --- | --- | --- |
|  |  |  |  |  |  |
| ***Furans*** |  |  |  |  |  |
| Furfural | 0.02-1 | y = 0.0001x + 0.0042 | 0.9955 | 15 | 50 |
| 2-Furanmethanol | 0.5-50 | y = 0.0001 x + 0.0164 | 0.9998 | 45 | 150 |
| 2(5*H*)-Furanone | 0.005-0.2 | y = 0.0028x - 0.0104 | 0.9997 | 1.7 | 5.7 |
| 5-Methylfurfural | 0.01-0.5 | y = 0.0005x - 0.0146 | 0.9984 | 6.9 | 23 |
| ***Pyridines*** |  |  |  |  |  |
| Pyridine | 0.01-0.5 | y = 0.0008x + 0.0223 | 0.9921 | 3.6 | 12 |
| 2,6-Dimethylpyridine | 0.005-0.2 | y = 0.0006x + 0.0038 | 0.9946 | 2.6 | 8.7 |
| 2,5-Dimethylpyrazine | 0.01-0.5 | y = 0.0004x + 0.0065 | 0.9963 | 3.8 | 13 |
| 2-Ethenylpyridine | 0.01-0.5 | y = 0.0009x + 0.0153 | 0.9961 | 3.8 | 13 |
| 3-Ethylpyridine | 0.002-0.5 | y = 0.0079x + 0.0063 | 0.9948 | 0.81 | 2.7 |
| 4-Ethenylpyridine | 0.02-1 | y = 0.0003x - 0.0083 | 0.9941 | 6.7 | 22 |
| 3-Ethenylpyridine | 0.02-1 | y = 0.0006x + 0.0002 | 0.9979 | 12 | 40 |
| 2,3,5-Trimethylpyrazine | 0.02-1 | y = 0.0004x + 0.0110 | 0.9954 | 6.4 | 21 |
| Nicotine | 0.05-1 | y = 0.00008 x - 0.0030 | 0.9990 | 15 | 50 |
| ***Others*** |  |  |  |  |  |
| Benzyl Alcohol | 0.05-1 | y = 0.00008 x - 0.00885 | 0.9772 | 25 | 84 |
| Linalool | 0.01-0.2 | y = 0.0004x - 0.0014 | 0.9961 | 5.8 | 19 |
| Menthol | 0.01-1 | y = 0.0002x + 0.0038 | 0.9975 | 6.7 | 22 |
| 4-Ethyl guaiacol | 0.01-1 | y = 0.0081x - 0.0678 | 0.9990 | 3.4 | 11 |
| Eugenol | 0.05-1 | y = 0.0003x - 0.0161 | 0.9995 | 16 | 53 |

Table S2

| No. | Compound | Retention time (min) | Precursor ion > Product ion (m/z) (Collision energy) |
| --- | --- | --- | --- |
| 1 | Pyridine | 3.4 | 79.00>52.10 (12) |
|  |  |  | 79.00>77.00 (36) |
| 2 | Furfural | 4.6 | 95.00>67.00 (6) |
|  |  |  | 96.00>54.00 (21) |
| 3 | 2-Furanmethanol | 5.0 | 98.00>70.10 (6) |
|  |  |  | 161.00>91.00 (15) |
| 4 | 2,6-Dimethylpyridine | 5.6 | 107.00>65.10 (21) |
|  |  |  | 107.00>92.10 (15) |
| 5 | 2,5-Dimethylpyrazine | 6.1 | 108.00>81.10 (9) |
|  |  |  | 108.00>67.10 (6) |
| 6 | 2(*5H*)-furanone | 6.4 | 84.00>55.00 (6) |
|  |  |  | 84.00>65.00 (21) |
| 7 | 2-Ethenylpyridine | 6.6 | 105.00>79.10 (9) |
|  |  |  | 79.00>52.10 (15) |
| 8 | 3-Ethylpyridine | 7.2 | 107.00>92.10 (12) |
|  |  |  | 107.00>65.10 (24) |
| 9 | 5-Methylfurfural | 7.3 | 109.00>53.10 (15) |
|  |  |  | 110.00>81.00 (12) |
| 10 | 4-Ethenylpyridine | 7.2 | 105.00>78.10 (15) |
|  |  |  | 105.00>52.10 (21) |
| 11 | 3-Ethenylpyridine | 7.4 | 105.00>78.00 (12) |
|  |  |  | 105.00>52.20 (21) |
| 12 | 2,3,5-Trimethylpyrazine | 8.4 | 122.00>81.10 (9) |
|  |  |  | 122.00>54.10 (18) |
| 13 | Benzyl Alcohol | 9.0 | 108.00>77.10 (27) |
|  |  |  | 108.00>79.00 (15) |
| 14 | Linalool | 10.7 | 93.00>77.10 (15) |
|  |  |  | 71.00>68.00 (36) |
| 15 | Menthol | 12.5 | 95.00>67.10 (9) |
|  |  |  | 71.00>67.00 (15) |
| 16 | 5-Hydroxy-2-methylpyridine | 12.5 | 109.00>80.10 (15) |
|  |  |  | 109.00>53.10 (27) |
| 17 | 4-Ethyl guaiacol | 15.2 | 137.00>94.10 (18) |
|  |  |  | 137.00>122.10 (12) |
| 18 | Nicotine | 16.8 | 162.00>84.10 (9) |
|  |  |  | 84.00>72.00 (15) |
| 19 | Eugenol | 17.1 | 164.00>149.10 (9) |
|  |  |  | 164.00>147.00 (24) |

Table S3

| IQOS Compound (µg/stick) | Regular | | |  | Menthol | | |
| --- | --- | --- | --- | --- | --- | --- | --- |
|  | CFP |  | Tenax GR |  | CFP |  | Tenax GR |
| ***Furans*** |  |  |  |  |  |  |  |
| Furfural | 1.6 ± 0.69 |  | 97 ± 15 |  | n.d. |  | 1.7 ± 0.17 |
| Furfuryl alcohol | 0.65 ± 0.48 |  | 14 ± 4.6 |  | n.d. |  | 0.11 ± 0.010 |
| 2(5H)-Furanone | 0.53 ± 0.15 |  | 2.4 ± 0.82 |  | n.d. |  | n.d. |
| 5-Methylfurfural | 0.43 ± 0.19 |  | 32 ± 6.6 |  | n.d. |  | 0.35 ± 0.03 |
| ***Pyridines*** |  |  |  |  |  |  |  |
| Pyridine | 0.15 ± 0.080 |  | 5.1 ± 0.75 |  | n.d. |  | 6.1 ± 0.85 |
| 2,6-Dimethylpyridine | n.d. |  | n.d. |  | n.d. |  | n.d. |
| 2,5-Dimethylpyrazine | 0.022 ± 0.010 |  | 0.75 ± 0.12 |  | n.d. |  | 0.014 ± 0.0021 |
| 2-Ethenylpyridine | n.d. |  | n.d. |  | n.d. |  | n.d. |
| 3-Ethylpyridine | n.d. |  | n.d. |  | n.d. |  | n.d. |
| 4-Ethenylpyridine | n.d. |  | 1.5 ± 0.29 |  | n.d. |  | 0.67 ± 0.14 |
| 3-Ethenylpyridine | 0.23 ± 0.046 |  | 0.26 ± 0.079 |  | n.d. |  | n.d. |
| 2,3,5-Trimethylpyrazine | n.d. |  | n.d. |  | n.d. |  | n.d. |
| ***Additives*** |  |  |  |  |  |  |  |
| Benzyl Alcohol | 0.017 ± 0.010 |  | 0.27 ± 0.072 |  | n.d. |  | 0.080 ± 0.010 |
| Linalol | 0.096 ± 0.020 |  | 0.42 ± 0.36 |  | n.d. |  | 0.020 ± 0.0 |
| Menthol | 0.57 ± 0.11 |  | 2.0 ± 0.28 |  | 0.95 ± 0.24 |  | 1000 ± 62 |
| 4-Ethyl guaiacol | n.d. |  | n.d. |  | n.d. |  | n.d. |
| Eugenol | n.d. |  | n.d. |  | n.d. |  | n.d. |
| ***Others*** |  |  |  |  |  |  |  |
| Nicotine (mg/stick) | 1.1 ± 0.043 |  | 0.13 ± 0.052 |  | 1.1 ± 0.080 |  | 0.20 ± 0.048 |

Table S4

| glo Compound (µg/stick) | Berry boost | | |  | Dark fresh | | |
| --- | --- | --- | --- | --- | --- | --- | --- |
|  | CFP |  | Tenax GR |  | CFP |  | Tenax GR |
| ***Furans*** |  |  |  |  |  |  |  |
| Furfural | 7.0 ± 1.4 |  | 160 ± 8.2 |  | 6.5 ± 1.1 |  | 170 ± 16 |
| Furfuryl alcohol | 1.5 ± 0.73 |  | 33 ± 4.5 |  | 1.3 ± 0.38 |  | 44 ± 5.5 |
| 2(5H)-Furanone | 0.31 ± 0.25 |  | 12 ± 1.9 |  | 0.15 ± 0.055 |  | 14 ± 1.1 |
| 5-Methylfurfural | 1.3 ± 0.24 |  | 54 ± 4.2 |  | 1.1 ± 0.23 |  | 69 ± 8.1 |
| ***Pyridines*** |  |  |  |  |  |  |  |
| Pyridine | 0.42 ± 0.17 |  | 2.3 ± 0.27 |  | 0.45 ± 0.15 |  | 2.6 ± 0.17 |
| 2,6-Dimethylpyridine | n.d. |  | n.d. |  | n.d. |  | n.d. |
| 2,5-Dimethylpyrazine | 0.019 ± 0.0048 |  | 0.40 ± 0.028 |  | 0.014 ± 0.0042 |  | 0.51 ± 0.0028 |
| 2-Ethenylpyridine | n.d. |  | n.d. |  | n.d. |  | n.d. |
| 3-Ethylpyridine | n.d. |  | n.d. |  | n,d |  | n.d. |
| 4-Ethenylpyridine | n.d. |  | 2.4 ± 0.24 |  | 0.038 ± 0.0048 |  | 0.90 ± 0.026 |
| 3-Ethenylpyridine | 0.030 ± 0.018 |  | 0.11 ± 0.028 |  | 0.066 ± 0.023 |  | 0.48 ± 0.089 |
| 2,3,5-Trimethylpyrazine | n.d. |  | n.d. |  | n.d. |  | n.d. |
| ***Additives*** |  |  |  |  |  |  |  |
| Benzyl Alcohol | 0.032 ± 0.0031 |  | 0.58 ± 0.12 |  | n.d. |  | 1.0 ± 0.054 |
| Linalol | 2.6 ± 0.34 |  | 10 ± 1.2 |  | n.d. |  | 0.050 ± 0.010 |
| Menthol | 360 ± 46 |  | 1300 ± 190 |  | 300 ± 17 |  | 1200 ± 45 |
| 4-Ethyl guaiacol | n.d. |  | n.d. |  | n.d. |  | n.d. |
| Eugenol | n.d. |  | n.d. |  | n.d. |  | n.d. |
| ***Others*** |  |  |  |  |  |  |  |
| Nicotine (mg/stick) | 0.90 ± 0.067 |  | 0.12 ± 0.035 |  | 1.0 ± 0.074 |  | 0.20 ± 0.028 |

Table S5

| ploom S Compound (µg/stick) | Regular taste | | |  | Menthol purple | | |
| --- | --- | --- | --- | --- | --- | --- | --- |
|  | CFP |  | Tenax GR |  | CFP |  | Tenax GR |
| ***Furans*** |  |  |  |  |  |  |  |
| Furfural | 0.10 ± 0.023 |  | 11 ± 0.48 |  | 0.017 ± 0.0046 |  | 2.0 ± 0.16 |
| Furfuryl alcohol | 0.035 ± 0.015 |  | 2.3 ± 0.16 |  | 0.025 ± 0.0026 |  | 2.1 ± 0.11 |
| 2(5H)-Furanone | 0.046 ± 0.021 |  | 1.4 ± 0.093 |  | n.d. |  | n.d. |
| 5-Methylfurfural | 0.013 ± 0.010 |  | 2.7 ± 0.16 |  | n.d. |  | 1.0 ± 0.074 |
| ***Pyridines*** |  |  |  |  |  |  |  |
| Pyridine | 0.012 ± 0.0012 |  | 0.73 ± 0.018 |  | n.d. |  | 0.40 ± 0.018 |
| 2,6-Dimethylpyridine | n.d. |  | n.d. |  | n.d. |  | n.d. |
| 2,5-Dimethylpyrazine | 0.0083 ± 0.0023 |  | 0.71 ± 0.023 |  | n.d. |  | 0.22 ± 0.028 |
| 2-Ethenylpyridine | n.d. |  | n.d. |  | n.d. |  | n.d. |
| 3-Ethylpyridine | n.d. |  | n.d. |  | n.d. |  | n.d. |
| 4-Ethenylpyridine | 0.027 ± 0.0022 |  | 0.62 ± 0.010 |  | n.d. |  | 1.1 ± 1.0 |
| 3-Ethenylpyridine | 0.026 ± 0.012 |  | 0.27 ± 0.013 |  | n.d. |  | 0.19 ± 0.060 |
| 2,3,5-Trimethylpyrazine | n.d. |  | n.d. |  | n.d. |  | n.d. |
| ***Additives*** |  |  |  |  |  |  |  |
| Benzyl Alcohol | 0.0085 ± 0.0065 |  | 0.21 ± 0.014 |  | n.d. |  | n.d. |
| Linalol | 0.0020 ± 0.00092 |  | 0.014 ± 0.0028 |  | 0.36 ± 0.051 |  | 3.0 ± 0.19 |
| Menthol | 0.12 ± 0.074 |  | 0.80 ± 0.15 |  | 88 ± 5.2 |  | 630 ± 12 |
| 4-Ethyl guaiacol | n.d. |  | n.d. |  | n.d. |  | n.d. |
| Eugenol | n.d. |  | n.d. |  | n.d. |  | 0.10 ± 0.01 |
| ***Others*** |  |  |  |  |  |  |  |
| Nicotine (mg/stick) | 0.65 ± 0.023 |  | 0.050 ± 0.0010 |  | 0.60 ± 0.057 |  | 0.030 ± 0.023 |

Table S6

| Compound (µg/stick) | 3R4F | | |
| --- | --- | --- | --- |
|  | CFP |  | Tenax GR |
| ***Furans*** |  |  |  |
| Furfural | 6.1 ± 0.64 |  | 80 ± 22 |
| Furfuryl alcohol | 1.5 ± 0.50 |  | 2.7 ± 0.73 |
| 2(5H)-Furanone | 3.0 ± 0.82 |  | 3.6 ± 0.57 |
| 5-Methylfurfural | 2.1 ± 0.53 |  | 10 ± 2.5 |
| ***Pyridines*** |  |  |  |
| Pyridine | 2.0 ± 0.75 |  | 31 ± 4.6 |
| 2,6-Dimethylpyridine | 0.14 ± 0.042 |  | 1.4 ± 0.30 |
| 2,5-Dimethylpyrazine | 0.41 ± 0.12 |  | 3.6 ± 0.63 |
| 2-Ethenylpyridine | 0.044 ± 0.010 |  | 0.54 ± 0.11 |
| 3-Ethylpyridine | 0.36 ± 0.13 |  | 1.0 ± 0.82 |
| 4-Ethenylpyridine | 0.30 ± 0.09 |  | 6.0 ± 0.87 |
| 3-Ethenylpyridine | 0.23 ± 0.11 |  | 3.5 ± 0.48 |
| 2,3,5-Trimethylpyrazine | n.d. |  | n.d. |
| ***Additives*** |  |  |  |
| Benzyl Alcohol | 0.33 ± 0.062 |  | 3.9 ± 0.35 |
| Linalol | 0.067 ± 0.018 |  | 0.057 ± 0.099 |
| Menthol | 1.1 ± 0.61 |  | 3.3 ± 3.3 |
| 4-Ethyl guaiacol | 0.42 ± 0.090 |  | 0.36 ± 0.10 |
| Eugenol | n.d. |  | n.d. |
| ***Others*** |  |  |  |
| Nicotine (mg/stick) | 1.4 ± 0.69 |  | 0.40 ± 0.078 |
